# Knockout of AMD-associated gene *POLDIP2* reduces mitochondrial superoxide in human retinal pigment epithelial cells

**DOI:** 10.18632/aging.204522

**Published:** 2023-02-16

**Authors:** Tu Nguyen, Daniel Urrutia-Cabrera, Luozixian Wang, Jarmon G. Lees, Jiang-Hui Wang, Sandy S.C. Hung, Alex W. Hewitt, Thomas L. Edwards, Sam McLenachan, Fred K. Chen, Shiang Y. Lim, Chi D. Luu, Robyn Guymer, Raymond C.B. Wong

**Affiliations:** 1 Centre for Eye Research Australia, Royal Victorian Eye and Ear Hospital, Melbourne, Victoria, Australia; 2 Ophthalmology, Department of Surgery, University of Melbourne, Melbourne, Victoria, Australia; 3 O’Brien Institute Department, St Vincent’s Institute of Medical Research, Melbourne, Victoria, Australia; 4 Departments of Surgery and Medicine, University of Melbourne, Melbourne, Victoria, Australia; 5 Menzies Institute for Medical Research, School of Medicine, University of Tasmania, Hobart, Tasmania, Australia; 6 Centre for Ophthalmology and Visual Science (Incorporating Lions Eye Institute), The University of Western Australia, Department of Ophthalmology, Royal Perth Hospital, Perth, Western Australia, Australia

**Keywords:** age-related macular degeneration, retina, CRISPR/Cas, mitochondria superoxide, POLDIP2

## Abstract

Genetic and epidemiologic studies have significantly advanced our understanding of the genetic factors contributing to age-related macular degeneration (AMD). In particular, recent expression quantitative trait loci (eQTL) studies have highlighted *POLDIP2* as a significant gene that confers risk of developing AMD. However, the role of *POLDIP2* in retinal cells such as retinal pigment epithelium (RPE) and how it contributes to AMD pathology are unknown. Here we report the generation of a stable human RPE cell line ARPE-19 with *POLDIP2* knockout using CRISPR/Cas, providing an *in vitro* model to investigate the functions of *POLDIP2*. We conducted functional studies on the *POLDIP2* knockout cell line and showed that it retained normal levels of cell proliferation, cell viability, phagocytosis and autophagy. Also, we performed RNA sequencing to profile the transcriptome of *POLDIP2* knockout cells. Our results highlighted significant changes in genes involved in immune response, complement activation, oxidative damage and vascular development. We showed that loss of *POLDIP2* caused a reduction in mitochondrial superoxide levels, which is consistent with the upregulation of the mitochondrial superoxide dismutase *SOD2*. In conclusion, this study demonstrates a novel link between *POLDIP2* and *SOD2* in ARPE-19, which supports a potential role of *POLDIP2* in regulating oxidative stress in AMD pathology.

## INTRODUCTION

Age-related macular degeneration (AMD), the most common cause of irreversible vision loss amongst people over 50 years old in developed countries [1, 2], is characterised by RPE degeneration and photoreceptor cell death. The RPE is critical to retinal homeostasis and some important roles include phagocytosis of photoreceptor outer segments, scavenging for damaged reactive oxygen species (ROS), and delivery of blood-derived nutrients to photoreceptors [3, 4]. Emerging evidence in recent years suggests that oxidative stress-induced mitochondrial damage in the RPE contributes to development of AMD [5–7].

Genome-wide association studies (GWAS) have contributed to our understanding of genetic associations to AMD and identified >60 single nucleotide polymorphisms (SNPs) implicated in AMD [8–10]. This included *CFH* and *ARMS2/HTRA1* loci that confer major susceptibility [11–13], which may affect the metabolomics in RPE cells [14]. In addition, other genomic methods have been developed to complement GWAS in identifying and confirming variants associated with diseases, such as expression quantitative trait loci (eQTL), transcriptome-wide association study (TWAS), and eCAVIAR [10, 15–17]. These techniques integrate genomic and transcriptomic data sets to confirm whether causal genes found in GWAS studies are driving disease association (reviewed in [18]). In particular, using eQTL and TWAS, a recent study highlighted *POLDIP2* at the *TMEM97/VTN* loci as a significant target gene associated with AMD [15]. However, the function of *POLDIP2* in the retina remains poorly understood. Our previous study has shown that *POLDIP2* is highly expressed in human RPE/choroid [19]. Unravelling the biological roles of *POLDIP2* in RPE is critical to advance our understanding of AMD pathogenesis.

*POLDIP2* encodes a multifunctional protein that localises in both the nucleus and the mitochondria [20]. Several studies have identified *POLDIP2* as a significant gene for AMD susceptibility [10, 15, 17] and it has been associated with vascular and neurodegenerative diseases [21, 22]. *POLDIP2* has been reported to play a role in a wide range of physiological and cellular processes [20]. Previous mouse studies showed that homozygous *Poldip2* knockout was embryonic lethal, *Poldip2*-/- embryos were significantly smaller than WT, and mouse embryonic fibroblasts (MEFs) exhibited reduced growth [23]. In addition, heterozygous knockout mice displayed lower levels of H_2_O_2_ production, which increased aortic extracellular matrix, increased vascular stiffness and impaired contractility, thus demonstrating that *Poldip2* expression is necessary for vascular structure and function [24]. Poldip2 has also been shown to be an oxygen-sensitive protein and regulates cell metabolism and mitochondrial function [25]. Poldip2 expression is downregulated by hypoxia and in cancer cells, leading to repression of lipoylation of the pyruvate and α-ketoglutarate dehydrogenase complexes and mitochondrial dysfunction. Interestingly, *POLDIP2* dysfunction has been implicated in Alzheimer’s disease, including metabolic and oxidative stress, neuroinflammation, as well as abnormal microvasculature and extracellular deposits [26, 27]. Overexpression of *POLDIP2* resulted in defective autophagy leading to increased Tau aggregation, whereas *POLDIP2* downregulation decreased ROS-induced Tau aggregation [22]. However, there is no previous study on the function of *POLDIP2* in the retina and its role in the development of AMD.

Recent advances in CRISPR technology offer exciting opportunities to manipulate genes and accelerate functional studies of AMD-associated genes. The combined use of Cas9 endonuclease and a single-stranded guide RNA (sgRNA) can target and cleave specific DNA sequences, thereby creating a double-stranded break and deletions (indels) to knockout genes [28]. Alternatively, a catalytically inactive Cas9 (dead Cas9, dCas9) can be coupled with a transcriptional repressor domain, such as Krupper-associate box (KRAB), to repress the expression of a target gene, termed CRISPR interference (CRISPRi) [29]. Together, these CRISPR/Cas9 systems provide useful tools to perform loss-of-function studies of AMD-associated genes in the RPE.

Using CRISPR/Cas9, here we report the generation of a human RPE cell line ARPE-19 with *POLDIP2* knockout. We showed that *POLDIP2* knockout resulted in upregulation in *SOD2* levels and decreased levels of mitochondrial superoxide. Also, our results highlighted the effect of *POLDIP2* loss on the transcriptome profile of RPE, and discovered upregulation of genetic signals related to immune response, oxidative damage and vascular development.

## RESULTS

### Evaluation of CRISPRi and CRISPR knockout of *POLDIP2* in ARPE-19

We first tested the use of CRISPRi to knockdown *POLDIP2* in ARPE-19, using an ARPE-19 cell line with stable expression of dCas9-KRAB we reported previously (ARPE-19-KRAB) [19]. To induce knockdown of *POLDIP2* expression, we designed 2 sgRNAs that target the proximity of the transcription start site (TSS) of the *POLDIP2* gene (Supplementary Figure 1, Supplementary Table 1). We transfected 2 different doses of sgRNAs, 360ng and 1000ng, into ARPE-19-KRAB. The efficiency of the 2 sgRNAs in knocking down *POLDIP2* expression was assessed using RT-qPCR. The results showed that sgRNA1 could not knockdown *POLDIP2* (360ng: 1.15±0.12 fold compared to mock; 1000ng: 1.15 fold compared to mock, Supplementary Figure 2), whereas sgRNA2 could repress *POLDIP2* expression level by ~24% (360ng: 0.77±0.05 fold compared to 1000ng: 0.76±0.15 fold compared to mock, Supplementary Figure 2). Since the CRISPRi-mediated knockdown levels observed were mild and likely insufficient for functional studies, next we tested the use of CRISPR/Cas9 to knockout *POLDIP2* in ARPE-19.

**Figure 1:**
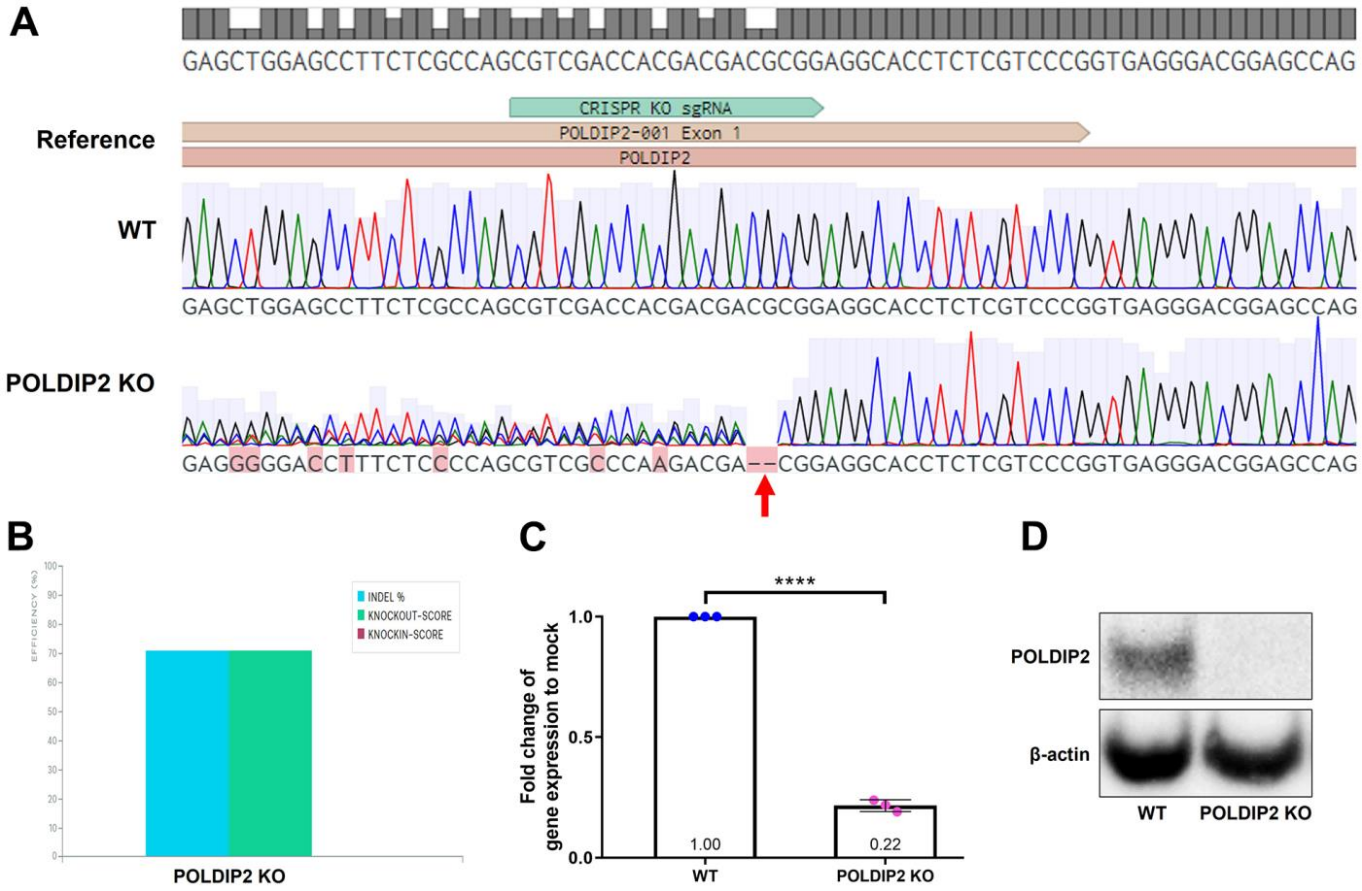
**Generation of POLDIP2 KO cell line.** (**A**) Sanger sequencing showed small indels in the coding sequence of *POLDIP2* in the POLDIP2 KO cell line, as indicated by the red arrow. (**B**) Quantification of indel percentage in knockout cell line compared to WT. (**C**) RT-qPCR analysis of *POLDIP2* repression using CRISPR KO. Values expressed as mean ± SEM, n=3. **** p<0.0001. (**D**) Western blot analysis of POLDIP2 protein repression.

To induce *POLDIP2* knockout, we transduced ARPE-19 with lentiviruses carrying sgRNA targeting the coding sequence of *POLDIP2* (Supplementary Figure 1). Following antibiotic selection, we generated a stable ARPE-19 cell line with *POLDIP2* knockout (POLDIP2 KO). POLDIP2 KO cells retained similar morphology to ARPE-19 wild type (WT) (Supplementary Figure 4). Critically, Sanger sequencing confirmed the presence of indels at the target site in *POLDIP2* CDS (Figure 1A), with a 70% indel percentage in POLDIP2 KO compared to WT (Figure 1B). We analysed the levels of *POLDIP2* gene expression using RT-qPCR and found a ~80% reduction of *POLDIP2* levels in POLDIP2 KO (0.22±0.01 compared to WT, Figure 1C). Also, western blot analysis showed an absence of POLDIP2 protein expression in POLDIP2 KO samples (Figure 1D). Finally, we performed a short tandem repeat (STR) analysis and confirmed that the knockout cell line originated from the parental ARPE-19 cell line (Supplementary Figure 3) [19]. Collectively, these results showed that we have successfully generated an ARPE-19 cell line with *POLDIP2* knockout.

### Functional studies of *POLDIP2* on ARPE-19 cells

**Figure 2:**
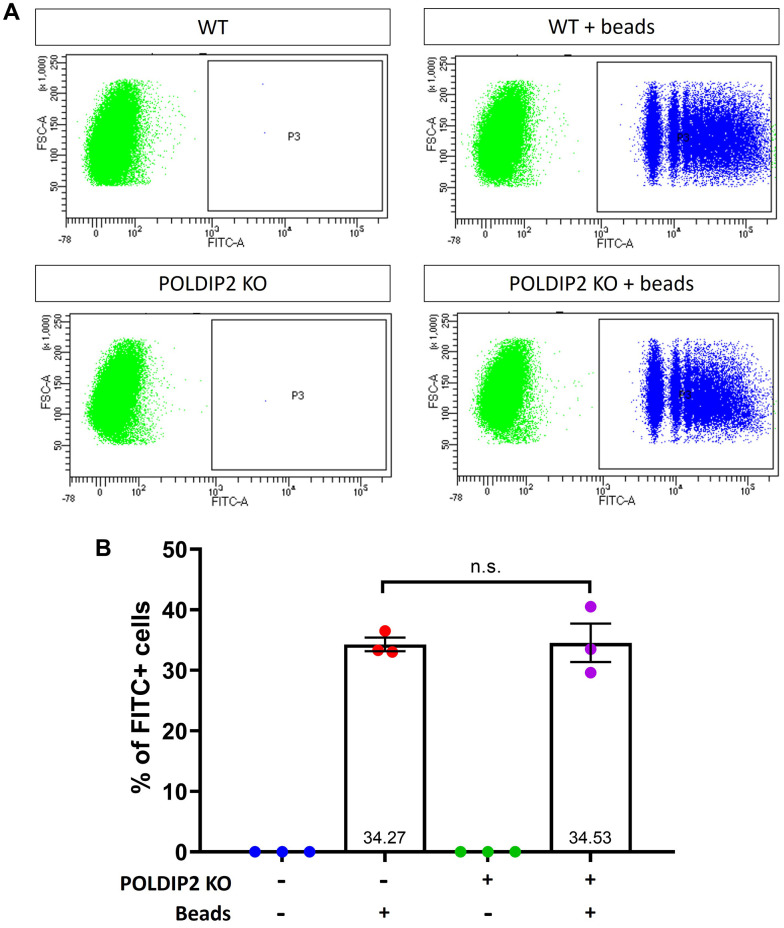
**POLDIP2 KO cells show normal levels of phagocytosis.** (**A**) Flow cytometry analysis of phagocytosis in WT and POLDIP2 KO treated with or without FITC+ fluospheres. (**B**) Pooled quantification results of n=3 biological repeats. Error bars represent SEM. n.s. not significant.

Phagocytosis is an important function of RPE to degrade ROS and maintain retinal homeostasis. Using POLDIP2 KO, we assessed the effect of *POLDIP2* on RPE phagocytosis. WT and POLDIP2 KO cells were incubated with FITC fluospheres and their phagocytosis ability was analysed by quantification of FITC+ cells using flow cytometry. Our results showed that the POLDIP2 KO cell line retained the ability to phagocytose FluoSpheres (Figure 2A). The proportion of FITC+ in POLDIP2 KO cells was 34.53±3.19%, compared to 34.27±1.12% in WT, which suggested the levels of phagocytosis between WT and POLDIP2 KO cells were similar (Figure 2B). Our results demonstrated that *POLDIP2* knockout did not affect the phagocytic ability of ARPE-19 cells.

**Figure 3:**
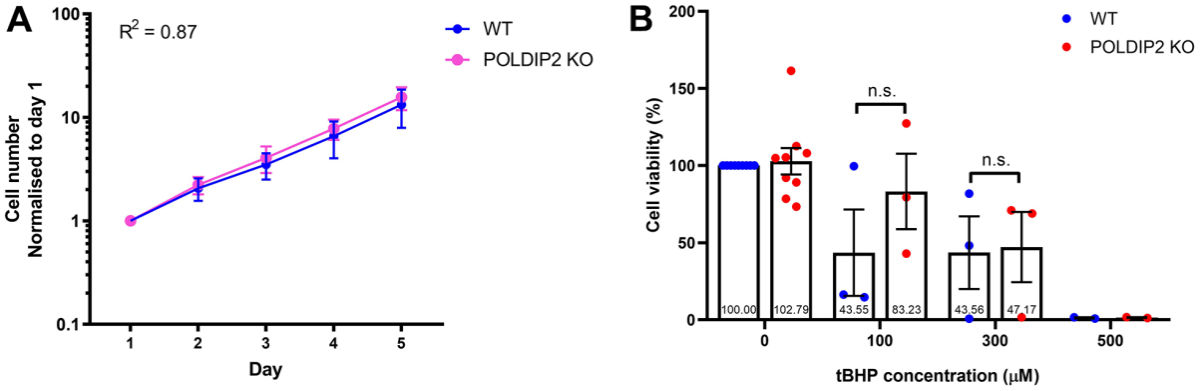
**Knockout of *POLDIP2* in ARPE-19 shows normal levels of cell proliferation and viability.** (**A**) Cell proliferation of WT and POLDIP2 KO cell lines. Normalised cell numbers expressed as mean ± SEM, n=3. (**B**) Cell viability analysis of WT and POLDIP2 KO cell lines in the presence or absence of tBHP. Results are presented as mean ± SEM of 2-9 biological repeats, each with 8 technical repeats.

Next, we investigated whether loss of *POLDIP2* would affect proliferation of ARPE-19. We showed that the POLDIP2 KO cell line showed a comparable growth rate to WT (POLDIP2 KO: R^2^=0.87; slope=3.47; WT: R^2^=0.87; slope=2.89, Figure 3A). Overall, our results showed that the effect of *POLDIP2* loss on cell proliferation is negligible in ARPE19.

Oxidative stress plays an important role in AMD pathogenesis and progression [30]. RPE has a high metabolic demand and thus mitochondria are a major source of ROS in the RPE. As a result, age-related mitochondrial dysfunction can induce oxidative stress in the RPE and contributes to AMD [31]. We assessed if *POLDIP2* knockout would affect cell viability of ARPE-19 in the presence of oxidative stress. To induce oxidative stress, we exposed the cells to tert-Butyl hydroperoxide (tBHP), a potent ROS-inducer commonly used to induce oxidative stress in cells and tissues. We then assessed cell viability of ARPE-19 treated with varying concentrations of tBHP. Our results showed that in the absence of tBHP, POLDIP2 KO cells showed a high level of cell viability and this level was comparable to WT (KO: 102.79±8.60% compared to WT, Figure 3B), which indicates that the loss of *POLDIP2* did not affect cell viability. Following treatment with 100μM of tBHP, POLDIP2 KO cells exhibited higher cell viability compared to WT (83.23±24.42% and 43.55±28.04% viability, respectively), albeit this difference is not statistically significant. 300μM of tBHP caused similar levels of cell death in POLDIP2 KO and WT cells (47.17±22,76% and 43.56±23.51% viability, respectively), while 500μM of tBHP killed most of the cells in POLDIP2 KO and WT (1.48±0.31% and 1.23±0.40% viability, respectively). Overall, our results showed that *POLDIP2* knockout did not significantly affect cell viability in the presence of oxidative stresses.

**Figure 4:**
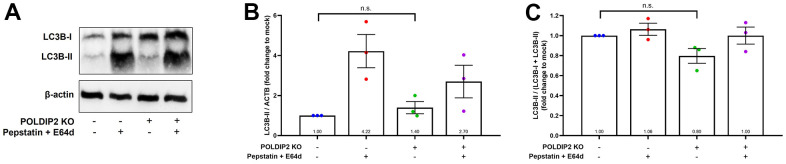
**Knockout of POLDIP2 did not significantly alter autophagy in ARPE-19.** (**A**) Western blot analysis of LC3B protein levels in WT and POLDIP2 KO samples. E64d and pepstatin A protease inhibitors (10 mg/ml each) were added where indicated. β-actin served as a loading control. (**B**) Quantification of the ratio of LC3B-II to β-actin. Values expressed as mean ± SEM, n=3 biological repeats. n.s. not significant. (**C**) Quantification of the ratio of LC3B-II/(LC3B-I + LC3B+II). Values expressed as mean ± SEM, n=3 biological repeats. n.s. not significant.

Induction of RPE autophagy has been considered as a therapeutic approach for AMD [32]. In this regard, a previous study showed that *POLDIP2* knockout increased autophagy in mouse embryonic fibroblasts [23]. Thus, we also investigated the role of *POLDIP2* in autophagy in ARPE-19 cells. We analysed LC3B levels as an indicator of autophagic flux [33] (Figure 4A). In the presence of lysosomal protease inhibitors pepstatin and E64d controls, which partially inhibit degradation of LC3B-II, LC3B-II levels increased in both WT (4.22±0.83 fold compared to mock, Figure 4B) and POLDIP2 KO samples (2.70±0.82 fold compared to mock). In basal condition, our results showed that LC3B-II levels in POLDIP2 KO cells were slightly higher than in WT (1.40±0.31 compared to WT, Figure 4B), albeit this difference is statistically insignificant. Similarly, LC3B-II/total LC3B ratio in POLDIP2 KO cells was slightly lower than in WT (0.80±0.07 compared to WT, Figure 4C), however this difference is also not statistically significant. Altogether, our results indicated that *POLDIP2* knockout did not significantly alter autophagic flux in ARPE-19.

### Transcriptome profiling of WT versus POLDIP2 KO cell lines

**Figure 5:**
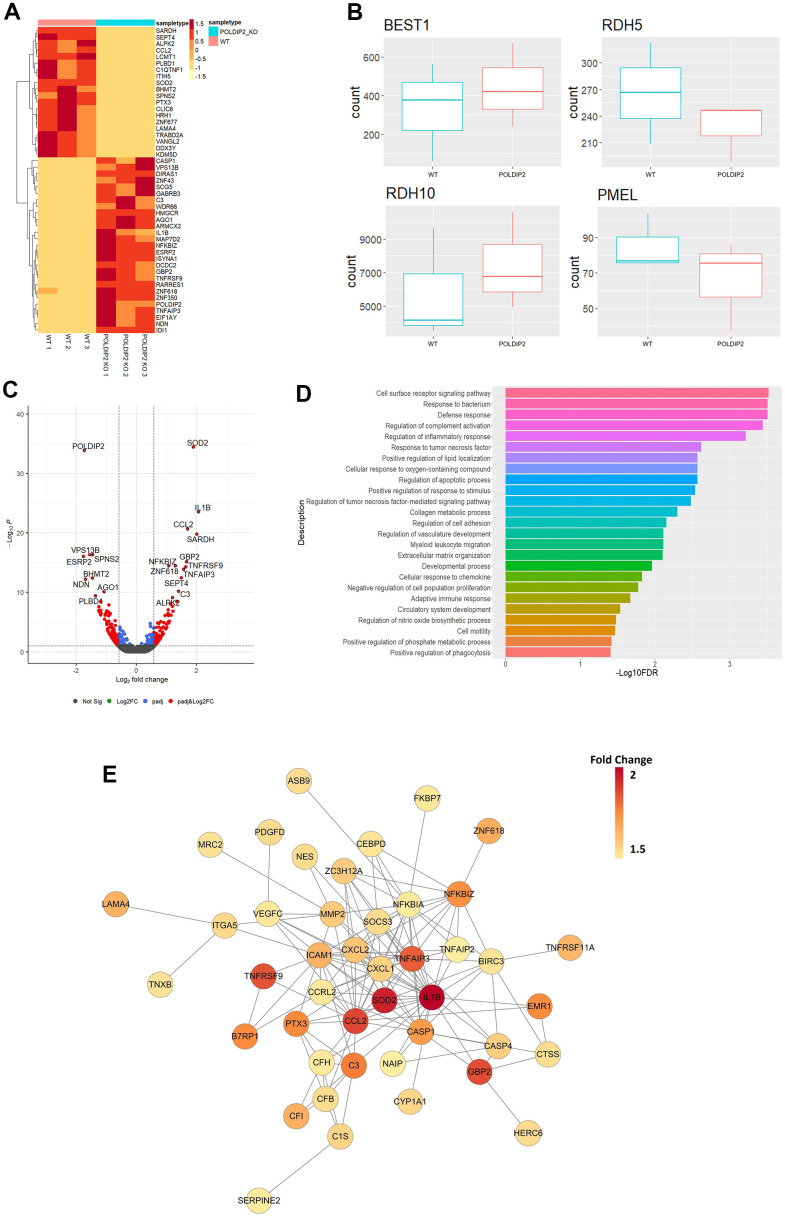
**Transcriptome profiling of POLDIP2 knockout in ARPE-19.** (**A**) Heatmap of top 50 DE genes detected in WT (n=3) and POLDIP2 KO (n=3) cell lines. (**B**) Boxplot of the expression levels of RPE markers BEST1, RDH5, RDH10, and PMEL in different samples. (**C**) Volcano plot of the top 20 DE genes labelled in POLDIP2 KO cell lines (n=3). (**D**) GO annotation of the top 50 up-regulated DE genes in POLDIP2 KO samples (n=3). (**E**) Network topology of the top up-regulated DE genes in POLDIP2 KO samples (n=3).

To investigate the impact of *POLDIP2* knockout on the RPE transcriptome profile, we performed RNA-seq on WT and POLDIP2 KO cell lines. Our results showed 93 upregulated genes and 203 downregulated genes in POLDIP2 KO compared to WT (Supplementary Data 1). Figure 5A illustrated the top 50 differentially expressed (DE) genes between POLDIP2 KO and WT. We compared the expression levels of four RPE markers *BEST1, PMEL, RDH5,* and *RDH10* between the two cell lines (Figure 5B). Notably, the expression levels of all four RPE markers between POLDIP2 KO and WT were similar, indicating that the ARPE-19 retained RPE identity following the loss of *POLDIP2*.

As expected, we observed that the most down-regulated gene was *POLDIP2*, which confirms the quality of the knockout cell line (Figure 5C). Interestingly, the most upregulated gene was *SOD2* (Figure 5C). *SOD2* encodes for a manganese superoxide dismutase (MnSOD), an antioxidant enzyme in the mitochondrial matrix that converts superoxide anion to hydrogen peroxide and protects mitochondria from oxidative stress [34]. Knockdown of *SOD2* in RPE of mice induced oxidative damage, which led to morphological abnormalities in RPE and Bruch’s membrane, as well as other changes associated with AMD such as increase in autofluorescence levels and bis-retinoid pigments located in RPE and drusen, and accumulation of oxidatively modified proteins [34]. Other top upregulated genes in POLDIP2 KO included *IL-1β, CCL2, SARDH, GBP2, NFKBIZ, TNFRSF9, TNFAIP3,* and *C3* (Figure 5C)*,* many of them are involved in the immune defense system.

To elucidate the biological roles of the DE genes, we performed gene ontology analysis using the top 50 upregulated DE genes in POLDIP2 KO samples (Figure 5D). Interestingly, loss of *POLDIP2* also upregulated several genes involved in the complement system, including *C3, IL1B, CFI, CFH, CFB* and *C1S*. Critically, *C3*, *IL1B*, *CFI*, *CFH*, and *CFB* have been identified as genes implicated in AMD development [11, 35–38]. In addition, our results highlighted that many upregulated DE genes are involved in the immune response, including chemokines (*CCL2, CXCL1, CXCL2*), cytokines (*IL1B*), and genes associated with cytokine-mediated signalling (*MMP2, ICAM1, GBP2, SOD2, NFKBIZ*), tumour necrosis factor-induced genes (*TNFRSF9, TNFAIP3*), and caspase cascade in apoptosis (*CASP1, CASP4*) (Figure 5E). Also, genes involved in vasculature development and homeostasis were upregulated following *POLDIP2* loss (*LAMA4*, *VEGFC*, *SOCS3*, and *ZC3H12A)*, as well as those involved in oxidative stress (*MMP2, ZC3H12A, SOD2,* and *TNFAIP3)*. Furthermore, network topology analysis revealed the inter-connectivity between these DE genes, such as complement system genes (*CFB*, *CFH*, *C3*, *C1S*, *CFI*), and caspase cascade genes (*CASP1, CASP4*) (Figure 5E). Collectively, our results suggested that loss of *POLDIP2* affected genes involved in a wide range of biological processes, including various aspects of the immune response such as complement activation, and AMD-related processes such as vasculature development and oxidative damage.

### *POLDIP2* knockout reduced mitochondrial superoxide in RPE cells

**Figure 6:**
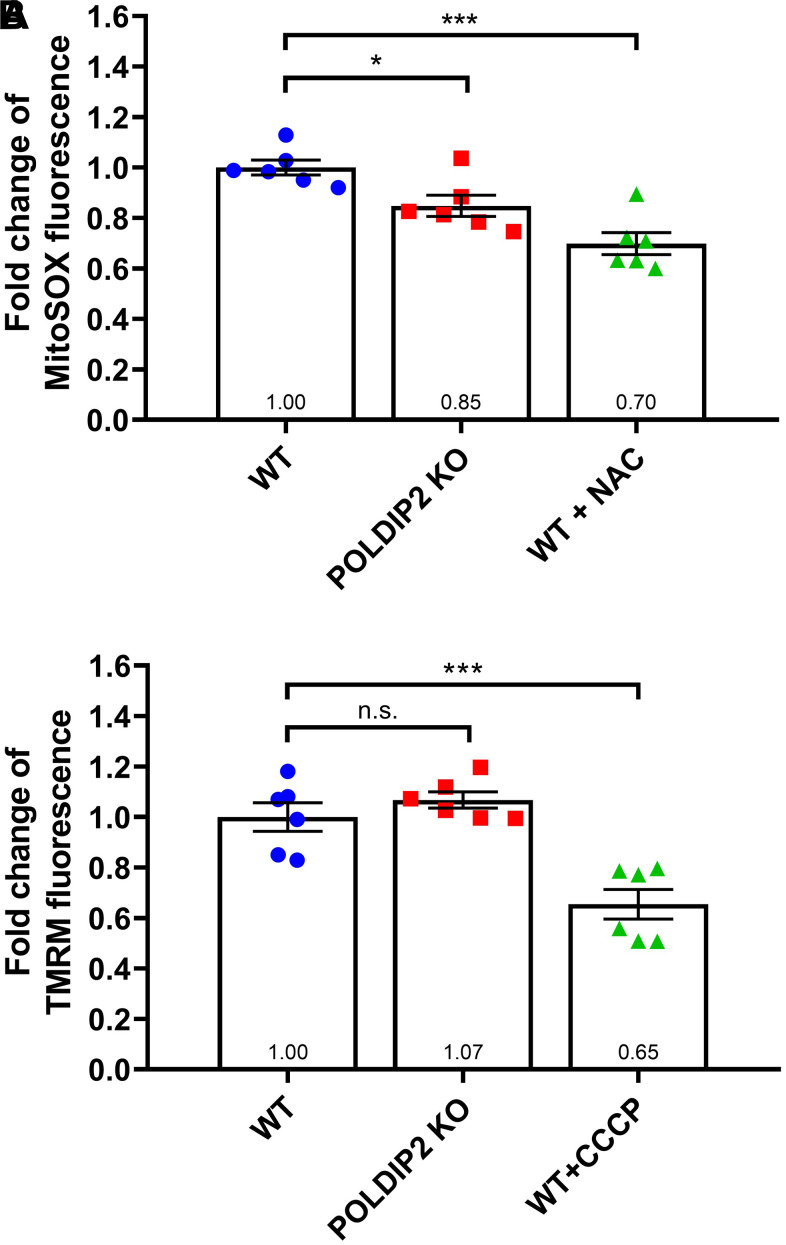
**POLDIP2 KO reduced mitochondrial superoxide.** (**A**) Analysis of mitochondrial superoxide levels in WT and POLDIP2 KO samples. MitoSox fluorescence is presented as fold change compared to WT control. N-acetyl-L-cysteine (NAC) was used as a positive control. (**B**) Analysis of mitochondrial membrane potential in WT and POLDIP2 KO samples. TMRM fluorescence is presented as fold change compared to WT control. Carbonyl cyanide 3-chlorophenylhydrazone (CCCP) was used as a positive control. Results are presented as mean ± SEM of 2 biological repeats, each with 3 technical repeats. n.s. not significant, * p<0.05 and *** p<0.001.

Given the mitochondrial gene *SOD2* was the most-upregulated gene in POLDIP2 KO, we further studied the role of *POLDIP2* in regulating mitochondrial oxidative stresses and activity. We performed a MitoSox assay to compare the levels of mitochondrial superoxide between WT and POLDIP2 KO cell lines. In the presence of the control N-acetyl-L-cysteine (NAC), which inhibited oxidation, MitoSox fluorescence decreased in WT samples as expected (0.70±0.04 compared to WT, Figure 6A). Interestingly, our analysis showed a significant reduction in mitochondrial superoxide levels in POLDIP2 KO (0.85±0.04 compared to WT, Figure 6A), which is consistent with the elevated expression of *SOD2* in POLDIP2 KO cells. In addition, we assessed the mitochondrial membrane potential using tetramethylrhodamine methyl ester (TMRM). Carbonyl cyanide 3-chlorophenylhydrazone (CCCP) was used as a control and reduced mitochondrial membrane potential in WT as expected (0.65±0.06 compared to WT, Figure 6B). Importantly, we observed similar levels of mitochondrial transmembrane potential between WT and POLDIP2 KO cells (POLDIP2 KO: 1.07±0.03 compared to WT, Figure 6B). These results indicate that mitochondrial membrane potential was not affected by the loss of *POLDIP2*. Altogether, our results identified a novel link of *POLDIP2* and *SOD2* in regulation of mitochondrial superoxide in RPE cells.

## DISCUSSION

Considerable effort has been made to understand the genetic factors that contribute to AMD using retinal cell models. However, there are significant limitations in studying AMD in animal models. For example, many disease-associated signs only develop in aged rodents, which increases the length of research time and subsequently the cost needed to house animals. Moreover, rats and mice do not have a macula and do not develop drusen or drusen-like deposits beneath the RPE [39]. In this sense, non-human primates are more appropriate animal models to study AMD, but are a lot more costly and require rigorous experiment setup [40]. In contrast, *in vitro* models offer a cheaper and easier alternative to facilitate studies of gene function in the retina. ARPE-19 is commonly used to study retinal cell biology and shows similar features to native RPE cells, such as expression of transporters, barrier formation, and phagocytic ability [41–43]. The present study reports the use of CRISPR for gene knockout in an *in vitro* human RPE model, as a model to study the function of an AMD-associated gene *POLDIP2*. Our first attempt to repress *POLDIP2* with a CRISPRi system yielded a low knockdown level; further optimisation of sgRNA would be important to improve this knockdown efficiency. Subsequently we moved to a CRISPR KO system and generated a stable ARPE-19 cell line with *POLDIP2* knockout, providing an important tool to study the effect of *POLDIP2* on biological processes relevant to RPE cells and to AMD pathophysiology.

A previous study showed that *Poldip2* affects growth rate and autophagy in MEFs [23]. *Poldip2* knockdown markedly reduced growth in MEFs. Further investigations revealed that *Poldip2-/-* MEFs were arrested or delayed in both G1 and G2/M phases of the cell cycle, and the protein levels of key cell cycle regulators also decreased following *Poldip2* knockdown. This study also reported an increase in autophagy following *Poldip2* knockout as indicated by higher levels of LC3B-II. However, we observed that loss of *POLDIP2* did not affect the growth rate and autophagic flux of ARPE-19 cells, suggesting that *POLDIP2* may have specific functions in different cell types.

To further study the roles of *POLDIP2*, we employed RNA-seq analysis to reveal gene expression changes caused by *POLDIP2* loss. We showed that a number of genes related to the immune system were upregulated in the POLDIP2 KO group. For instances, *IL-1β* was the second most upregulated gene in POLDIP2 KO samples. *IL-1β* is transcribed following pro-inflammatory stimuli [44] and its expression is increased in the central nervous system in acute and chronic neurodegenerative diseases [45]. In light-challenged mice, a model of dry AMD, subretinal mononuclear phagocytes expressed IL-1β, which induced rod death and cone segment loss [36, 46]. *CCL2* is another gene that was highly upregulated in POLDIP2 KO cells. CCL2 is a chemokine that directs leukocyte migration and its expression in RPE and the retina is very low in healthy young adult animals [47], but is elevated with ageing [47], following acute inflammation [48, 49], and oxidative insult in the RPE [50]. GBP2 is a member of the large GTPase superfamily that is strongly induced by interferon-γ (IFN-γ) and its expression in the retina was found to be significantly increased in aged and adult rats compared to young rats [51]. Tumour necrosis factors *TNFRSF9* and *TNFAIP3* were also upregulated following *POLDIP2* knockout. These genes are associated with dendritic cell maturation, among which reduced levels of *TNFAIP3* was found to enhance dendritic cell function in patients with AMD [52, 53]. Our analysis also showed increased expression of the complement component *C3*, which is strongly associated with AMD [35, 54] and has been shown to be interconnected with the expression of VEGF, RPE deterioration, geographic atrophy, and development of choroidal neovascularization [55–57].

Our findings revealed that following tBHP-induced oxidative stress, *POLDIP2* knockout did not lead to any remarkable changes to ARPE-19 viability. It has been reported that *Poldip2* silencing in MEFs increases cell sensitivity to oxidative stress, as indicated by cell viability assay after H_2_O_2_ treatment [58]. While we observed normal cell viability in POLDIP2 KO cells following tBHP-induced oxidative stress in this present study, future studies could look at the effect of *POLDIP2* using other oxidative stress assays. Interestingly, we identified a link between *POLDIP2* KO and the reduced levels of mitochondrial superoxide. Our results supported that the loss of *POLDIP2* upregulated *SOD2*, which enhanced the conversion of superoxide anion to hydrogen peroxide and resulted in lower superoxide levels in the mitochondria. Previous studies have also demonstrated that *POLDIP2* is involved in oxidative signalling through cellular oxidases. Poldip2 has been reported to be an upstream regulator of the NADPH oxidase Nox4 [59, 60], an enzyme functionally linked to proinflammatory responses, oxygen sensing, and senescence. In vascular smooth muscle cells, Poldip2 upregulates endogenous reactive oxygen species via Nox4 and positively regulates basal ROS production [59]. In addition, Poldip2 also mediates oxidative stress and inflammation via interaction with Nox4 in lung epithelial cells and downregulation of Poldip2 leads to decreased production of ROS [60]. It was demonstrated that Poldip2 deficiency protects against lung edema and vascular inflammation through suppressing mitochondrial ROS in a mouse model of acute respiratory distress syndrome [61]. Furthermore, deletion of *Sod2* in mice has been shown to disrupt RPE morphology, reduce RPE function, and elevate oxidative stress in RPE [6]. Another study showed that treatment with citicoline can reduce oxidative stress in AMD RPE cybrid cells [62]. These studies support the notion that oxidative stresses regulated by *POLDIP2* and *SOD2* play a role in RPE dysfunction and progression of AMD.

There are limitations to this study. The current study identified a link between *POLDIP2* and *SOD2* in one human RPE model only - the ARPE-19. Although ARPE-19 has morphological and functional features of human RPE, there are limitations to its use as a RPE model, including reduced levels of some RPE markers and low transepithelial resistance compared to native RPE [63]. The loss of RPE melanin has been reported in AMD [64], and since ARPE-19 cells do not retain the original pigmented phenotype of the RPE, this hinders an opportunity to study changes in melanin pigment in RPE. Other *in vitro* RPE models have been reported, including human primary RPE cells, pluripotent stem cells-derived RPE cells (reviewed in [18] and [65]). Future studies using primary RPE cells or pluripotent stem cells-derived RPE would provide a suitable model to further validate the functions of *POLDIP2* in human RPE cells. The use of 3D *in vitro* models, such as retinal organoids derived from pluripotent stem cells, would also provide an interesting opportunity to study the functional role of *POLDIP2* in relation to the cellular interaction within the retinal macroenvironment. Moreover, a recent study has generated an improved *Poldip2* knockout mouse model [66], further research focusing on the retina of these knockout mice would provide valuable knowledge to understand the role of *Poldip2* in retinal development and functions.

In summary, we have generated a *POLDIP2* knockout ARPE-19 cell line using CRISPR/Cas9 and studied the biological functions of *POLDIP2*. To our knowledge, this is the first functional study of *POLDIP2* in retinal cells to understand its potential role in AMD. The POLDIP2 KO cell line possesses normal proliferation, phagocytosis, autophagy, sensitivity to oxidative stress-induced cell death and mitochondrial activity. Interestingly, we identified a novel link between *POLDIP2* and mitochondrial oxidative stress modulation via *SOD2*, supporting a potential role for *POLDIP2* in AMD pathogenesis. Future studies to investigate the precise mechanism by which *POLDIP2* regulates oxidative stress signalling pathways would be important to advance our understanding of AMD genetics.

## MATERIALS AND METHODS

### Cell culture

HEK293FT, ARPE-19, ARPE-19-KRAB, and POLDIP2 KO cells were maintained at 37° C and 5% CO2 in a culture medium containing DMEM (Thermo Fisher) supplemented with 10% [v/v] Fetal Bovine Serum (FBS), 2mM GlutaMAX, and 0.5% Penicillin-Streptomycin (all from Thermo Fisher). Cells were passaged before they reached confluency using 0.25% Trypsin-EDTA (Thermo Fisher).

### Generation of *POLDIP2* knockout ARPE-19 cell line

For construction of a lentiviral vector co-expressing SpCas9 and target sgRNA, the complementary DNA oligos of the sgRNAs targeting *POLDIP2* were commercially synthesised (Supplementary Table 1), then phosphorylated and annealed using T4 Ligation Buffer and T4 PNK (NEB) to form the double-stranded DNA using the following thermal parameters: at 37° C for 30 min, and at 95° C for 5 min, followed by decreasing at 5° C/min to 25° C. The lentiviral vector lentiCRISPRv2 (Addgene, #52961) was linearised by BsmBI and dephosphorylated using CIP (NEB). The product was purified by gel electrophoresis and gel extract. The annealed DNA oligos of the sgRNA was ligated to the linearised lentiCRISPRv2 vector by T4 DNA ligase (NEB).

For lentivirus generation, 7x10^6^ HEK293FT cells were seeded in a 10 cm^2^ dish one day prior to transfection, cultured in Opti-MEM supplemented with 5% FBS and 200 μM sodium pyruvate (all from Thermo Fisher). Lentivirus was generated using the 3^rd^ generation packaging system. The transfer plasmid and three packaging vectors pMDLg/pRRE (Addgene, #12251), pRSV-Rev (Addgene, #12253), and pMD2.G (Addgene, #12259) were transfected into HEK293FT cells using Lipofectamine 3000 (Thermo Fisher). 6 hours after transfection, the medium containing Lipofectamine 3000 was replaced with fresh media. The supernatant containing the virus was collected 48 and 72 hours after transfection, and subsequently filtered (0.45 μm filter, Sartorius) and concentrated using PEG-it precipitation solution (SBI Integrated Sciences) according to the manufacturer's instructions. The viral titre was determined using Lenti-X p24 Rapid Titre Kit (Takara Bio) according to manufacturer’s instructions.

ARPE-19 cells were transduced with the *POLDIP2* knockout lentiviruses (MOI=10) overnight, followed by selection with 2μg/ml puromycin 3 days after transduction. Transduced ARPE-19 cells were further expanded to obtain the POLDIP2 KO cell line.

### RT-qPCR analysis

RNA extraction was performed using the illustra RNAspin Mini Kit (GE Healthcare Life Sciences) according to manufacturer’s instructions. RNA concentration and quality were measured using NanoDrop. cDNA was synthesised using the High-capacity cDNA reverse transcription kit with RNase inhibitor (Thermo Fisher). RT-qPCR reaction mixture was set up using TaqMan Fast Advanced Master Mix (Thermo Fisher) and Taqman probes for *POLDIP2* (Hs00210257_m1) and the housekeeping gene β-actin (Hs99999903_m1) (Thermo Fisher). RT-qPCR was performed on the 7500 Fast or StepOnePlus Real-Time PCR System (Thermo Fisher), following manufacturer’s instructions. The Delta-Delta Ct method was used to calculate and compare relative mRNA levels to control.

### Cell proliferation assay

Cell proliferation was analysed by cell count over 5 days. On day 0, 1.2x10^4^ ARPE-19 WT and POLDIP2 KO cells were seeded in a well of a 24-well plate. Cells from three wells per cell line were harvested daily for the next five days using 0.25% Trypsin-EDTA, stained with trypan blue (Thermo Fisher), and counted using the Countess Automated Cell Counter (Thermo Fisher). The average number of live cells was calculated for each day and normalised to day 1 cell number.

### Cell viability assay

Cell viability assay was performed using a CellTiter-Glo Luminescent Cell Viability Assay (Promega) following the manufacturer's instructions. On day 0, 10^4^ ARPE-19 and POLDIP2 KO cells were seeded in a well of a 96-well plate. On day 2, the cells were treated with various concentrations of tert-butyl hydroperoxide (tBHP) (Sigma). On Day 3, the media was replaced with 25μl of fresh media and 25μl of CellTiter-Glo Reagent was added to each well. The plate was incubated at room temperature for 10 minutes to generate a luminescent signal. 40μl of cell lysate from each well was loaded to an opaque white luminometer plate and luminescence was recorded using a Spark 20M microplate reader (Tecan). The OD reading was normalised to the control condition (WT without tBHP).

### Immunoblotting

Protein levels were assessed by western blot analysis. Cells were lysed using RIPA Buffer (Thermo Fisher) and sonicated. Protein concentrations were determined using Pierce BCA Protein Assay Kit (Thermo Fisher). Protein lysates were mixed with 4X Laemmli Sample Buffer (Biorad, #1610747) and 2-Mercaptoethanol (1:40, Sigma) and heated at 95° C for 5 minutes. Proteins were separated via 15% SDS-PAGE gels and transferred to PVDF membranes. Following blocking, the immunoblots were incubated overnight with primary antibodies: anti-beta actin (1:2500, Abcam), anti-LC3B (1:1000, Cell Signaling), and anti-POLDIP2 (1:1000, Abcam). Membranes were then incubated with secondary antibodies: goat anti-rabbit IgG HRP or goat anti-mouse IgG HRP (1:2500, all from Abcam). Bands were visualised using Pierce ECL Western Blotting Substrate (Thermo Fisher) using a BioRad Chemidoc MP Imaging System.

### Phagocytosis assay

Phagocytosis analysis was performed using fluorescent microspheres. 4x10^5^ cells were seeded in a well of a 6-well plate. Cells were incubated with 1μm diameter, yellow-green (505/515 nm) carboxylate-modified microspheres (FluoSpheres, Thermo Fisher) at a quantity of 160 beads per cell for 4 hours. Cells were dissociated with 0.25% Trypsin-EDTA, washed with DPBS + 1% FBS 5 times, and resuspended in 400μl of DPBS + 1% FBS. The cell samples were added with 0.1 μg/ml of DAPI (Sigma) and passed through a strainer (*In Vitro* Technologies). Quantification of FITC+ cells was performed using a BD LSRFortessa Cell Analyzer (BD Biosciences). Gating was set with a negative control using WT cells without FluoSpheres.

### Mitochondrial membrane potential assay

Mitochondrial membrane potential was assessed using tetra-methyl rhodamine methyl ester (TMRM), which selectively accumulates in the mitochondria according to the mitochondrial membrane potential. Cells were incubated with a non-quenching dose of TMRM at 10 nM in culture media. The mitochondrial respiratory uncoupler Carbonyl cyanide 3-chlorophenylhydrazone (CCCP, 50 mM), was used as a positive control to dissipate the mitochondrial membrane potential. Images were captured at 200x magnification with a fluorescence microscope (Olympus IX71) and the total corrected cell fluorescence was assessed using ImageJ. At least 600 cells from 3 random fields were counted per group.

### Mitochondrial superoxide production assay

Mitochondrial production of reactive oxygen species (ROS) was assessed using MitoSOX Red (Thermo Fisher). Cells treated with 5 mM of the antioxidant N-acetyl-L-cysteine (Sigma-Aldrich) were used as a positive control. Images were captured at 200x magnification with a fluorescence microscope (Olympus IX71) and the total corrected cell fluorescence was assessed using ImageJ. At least 600 cells from 3 random fields were counted per group.

### RNA sequencing

Total RNA of the ARPE-19 cell lines was extracted using the Illustra RNAspin Mini Kit (GE Healthcare Life Sciences) according to manufacturer’s instructions. RNA quality was checked by bioanalyzer and the TruSeq Stranded mRNA kit (Illumina) was used to prepare transcriptome libraries. The libraries were sequenced using Illumina Novaseq 6000 100bp single-end sequencing, at a depth of 38-50 million reads per sample (Australian Genome Research Facility).

Following the abundance estimates of transcripts generated by *Salmon* v1.8, the pseudocounts were mapped to the GRCh38 genome assembly using the *tximport* v1.22.0 package [67]. The gene count matrix was inputted as an DESeq2Dataset object using the *DESeqDataSetFromTximport* () function, then the DESeq2Dataset object was normalised using the *counts()* function to make fair gene expression comparisons between samples [68]. The normalised dataset was analysed with the *DESeq2* v1.34.0 package using rlog transformation. The sample-level QC was performed using principal components analysis while the gene-level QC was performed using hierarchical clustering. For differential expression analysis, the significant differentially expressed genes were determined using the *filter* () function with adjusted p value of < 0.05 and fold change > 1.5. The expression data of significant differentially expressed genes was visualised using the *ggplot2* v3.3.6, *pheatmap* v1.0.12 and *EnhancedVolcano* v1.12.0 R package [69–71]. The upregulated DE genes were used for network topology analysis using Cytoscape v3.8 [72], with default setting of full STRING network, a confidence score cutoff of 0.4 and no additional interactor, resulting in a network with 44 DE genes. Gene ontology analysis was performed for the upregulated DE genes using functional enrichment analysis in Cytoscape with default settings.

### Short tandem repeat analysis

Genomic DNA of ARPE-19 cell lines was extracted using the Wizard SV Genomic DNA Purification System (Promega), following manufacturer’s instructions. Short tandem repeat analysis was performed using the GenePrint 10 system (Promega) by the Australian Genome Research Facility.

### Statistical analysis

RT-qPCR for *POLDIP2* expression, phagocytosis, cell viability, and autophagy assays were assessed using unpaired t-test, mitochondrial assays were analysed using one-way ANOVA (GraphPad Prism). p<0.05 is used to establish statistical significance.

### Data availability

The transcriptome data generated in this study are available in the NCBI Gene Expression Omnibus database (GSE207158), including raw data, processed data, information of the experimental design, sequencing and processing pipeline.

## Supplementary Material

Supplementary Figures

Supplementary Table 1

Supplementary Data 1
