# Supplementary material for: Knockout of AMD-associated gene POLDIP2 reduces mitochondrial superoxide in human retinal pigment epithelial cells
Source: Aging (Albany NY). 2023 Feb 16;15(6):1713–33. doi: 10.18632/aging.204522 (PMC10085620; doi:10.18632/aging.204522)
Supplement: Supplementary Figures [file aging-15-204522-s001.pdf]

SUPPLEMENTARY FIGURES

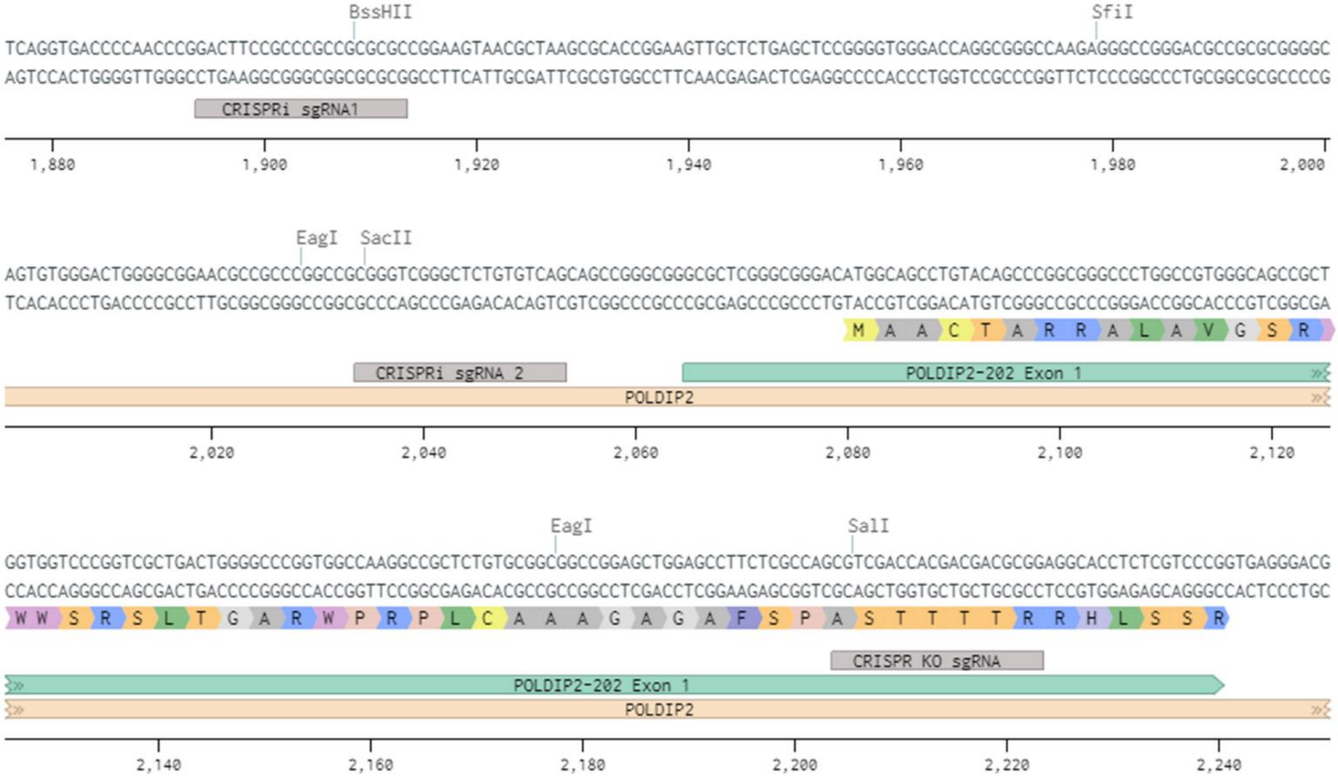

Supplementary Figure 1. Schematic diagram of the 5' region of human *POLDIP2* gene with sgRNA target areas near the transcription start site (TSS). CRISPRi sgRNA1 and CRISPRi sgRNA2 were used for *POLDIP2* knockdown and CRISPR KO sgRNA was used for *POLDIP2* knockout.

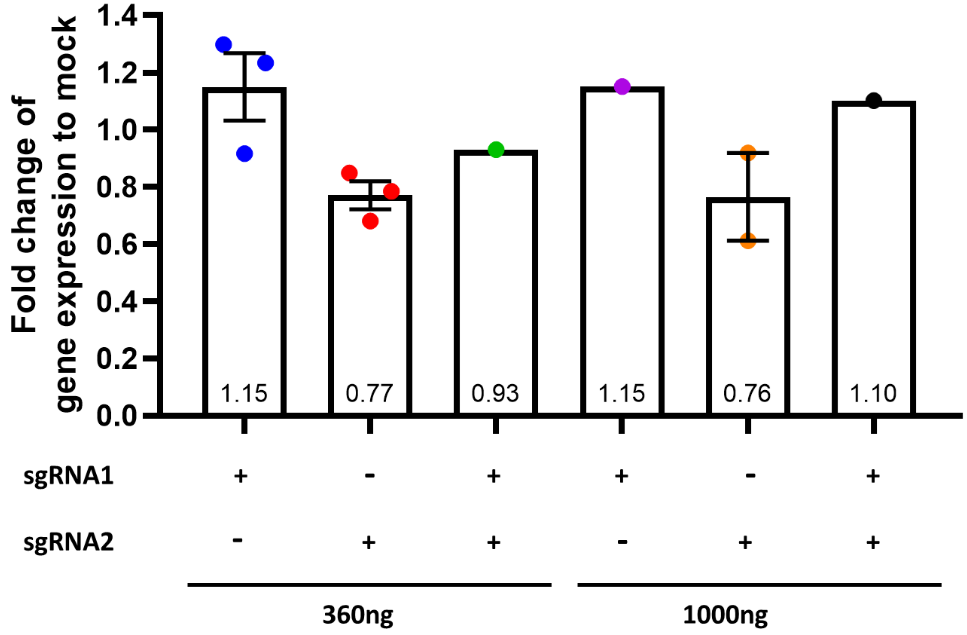

Supplementary Figure 2. Testing *POLDIP2* repression in ARPE-19-KRAB cell line. Cells were analysed using RT-qPCR 3 days after transfection with the indicated sgRNAs. Values were normalised to a ARPE-19 mock control and expressed as mean ± SEM.

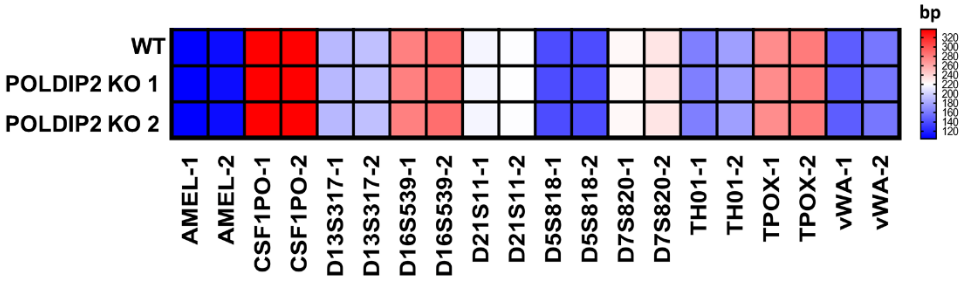

Supplementary Figure 3. Heatmap of short tandem repeat analysis of 10 polymorphic markers of WT [19] and POLDIP2 KO cell lines. Allele 1 and 2 are designated as -1 and -2 respectively.

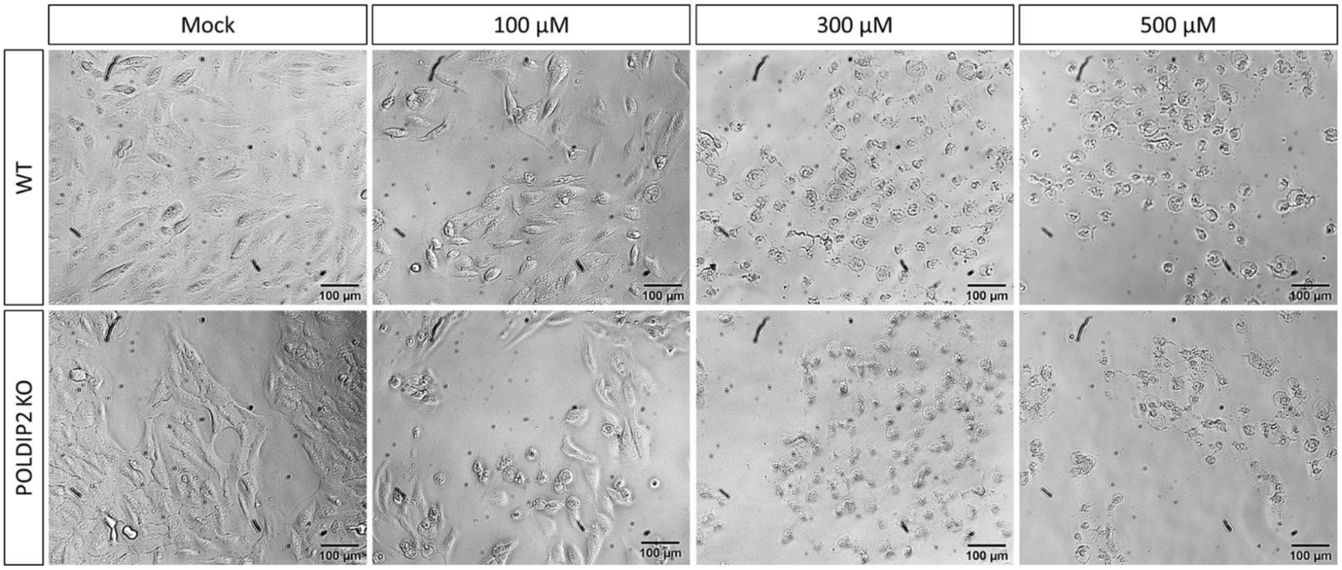

Supplementary Figure 4. Representative images of WT and POLDIP2 KO cells after 3 days of tBHP treatment with various concentrations (100 μM, 300 μM, and 500 μM) and control (Mock).
