# Supplementary material for: Knockout of AMD-associated gene POLDIP2 reduces mitochondrial superoxide in human retinal pigment epithelial cells
Source: Aging (Albany NY). 2023 Feb 16;15(6):1713–33. doi: 10.18632/aging.204522 (PMC10085620; doi:10.18632/aging.204522)
Supplement: Supplementary Table 1 [file aging-15-204522-s002.pdf]

SUPPLEMENTARY TABLE

Supplementary Table

Supplementary Table 1. Information of sgRNAs used in this study.

Name	TSS distance	Strand	Sequence	PAM	On-target score	Off-target score
POLDIP2 CRISPRi sgRNA1	151	-	GACTTCCGCCCGCCGCGCGC	CGG	34.5	83.4
POLDIP2 CRISPRi sgRNA 2	11	-	CTGACACAGAGCCCGACCCG	CGG	64.0	49.0
POLDIP2 CRISPR KO	139	+	CGTCGACCACGACGACGCGG	AGG	68.2	93.1

TSS distance is based on the transcription start site defined by Ensembl. On-target and off-target scores are based on [1].

Supplementary Reference

1. Doench JG, Fusi N, Sullender M, Hegde M, Vaimberg EW, Donovan KF, Smith I, Tothova Z, Wilen C, Orchard R, Virgin HW, Listgarten J, Root DE. Optimized sgRNA design to maximize activity and minimize off-target effects of CRISPR-Cas9. Nat Biotechnol. 2016; 34:184–91. https://doi.org/10.1038/nbt.3437 PMID:26780180
